# Kinetic Modeling of Phosphate Adsorption by Preformed and In situ formed Hydrous Ferric Oxides at Circumneutral pH

**DOI:** 10.1038/srep35292

**Published:** 2016-10-14

**Authors:** Yanpeng Mao, Qinyan Yue

**Affiliations:** 1 National engineering laboratory for coal-fired pollutants emission reduction, School of Energy and Power Engineering, Shandong University, Jinan 250100, PR China; 2 School of Environmental Science and Engineering, Shandong University, Jinan 250100, PR China

## Abstract

Kinetics of phosphate removal by Fe(III) was investigated by both preformed and *in situ* formed hydrous ferric oxides (HFO) at pH 6.0–8.0. A pseudo-second-order empirical model was found to adequately describe phosphate removal in the two cases. The Elovich and intra-particle diffusion models, however, were only capable of describing phosphate adsorption to preformed HFO (PF-HFO). By using surface complexation kinetic models (SCKMs) to describe phosphate adsorption to PF-HFO, the adsorption rate constant (0.0386–0.205 mM^−1^ min^−1^ for SCKM-1 and 0.0680–0.274 mM^−1^ min^−1^ for SCKM-2) decreased with increasing pH while the protonation reaction rate constant in SCKM-2 (0.0776–0.0947 mM^−1^ min^−1^) increased over the pH range 6.0–8.0. Using the rate constants obtained from the process of phosphate adsorption to PF-HFO, the amount of active surface sites on the *in situ* formed HFO were calculated as 0.955 ± 0.170, 1.46 ± 0.39 and 2.98 ± 0.78 mM for pH = 6.0, 7.0 and 8, respectively. Generally, as the SCKMs incorporate phosphate complexation on HFO surface sites and protons competiting for the surface sites, they could provide a good description of the rate and extent of phosphate removal by both preformed and *in-situ* formed HFO over a wide range of conditions.

To achieve high-efficiency phosphate removal from wastewaters, chemical precipitation of phosphorus using salts of aluminum (such as aluminum sulfate), iron (such as ferric chloride) or calcium (such as lime) is normally used^1^,^2^,^3^,^4^. Ideally, to removal phosphate by Fe(III), the most effective route is to form FePO_4_(s), in which the formation of a solid possesses a 1:1 molar ratio of Fe and P. However, the rate constants for formation of FePO_4_(s) at pH 6.0–8.0 are at least two orders of magnitude lower than the rate constants for Fe(III) hydrolysis and precipitation over this pH range (Mao *et al*., unpublished). As such, the phosphate removal mechanism on addition of Fe(III) salts is unlikely to involve the precipitation of FePO_4_(s) but, rather, the formation of hydrous ferric oxides (HFO) followed by phosphate adsorption. Additionally, Galarneau and Gehr^5^ confirmed that phosphate removal using Al(III) salts involves production of Al(OH)_3_(s), followed by phosphate adsorption to this oxyhydroxide. Smith *et al*.^6^ have made the case that phosphate removal by Fe(III) salts is likely to be dominated by phosphate adsorption to HFO rather than precipitation of the more soluble FePO_4_(s).

While the rate of formation of HFO at circumneutral pH is recognized to be rapid, the rate (and extent) of phosphate adsorption by HFO has been shown to be strongly dependent on a variety of factors including the age or crystallinity of the iron oxide and the presence of competing anions and sorbing substrates such as natural organic matter^7^. Extensive research has been published on the kinetics of phosphate adsorption by stable crystalline iron oxides or modified iron oxides including goethite^8^,^9^, iron hydroxide-eggshell waste^10^, activated carbon loaded with Fe(III) oxide^11^, ferric sludge^12^ and iron oxide tailings^13^. However, there has been surprisingly little work on the kinetics of phosphate adsorption by freshly formed hydrous ferric oxide. Shang *et al*.^14^ examined and modeled phosphate adsorption by short-range ordered aluminum and iron precipitates, while Crosby *et al*.^15^ investigated phosphate adsorption by iron oxyhydroxides. However, neither the adsorption mechanism nor the effect of pH on the kinetics of phosphate adsorption to HFO were clearly elucidated in these studies.

There have been many publications dealing with phosphate adsorption kinetics, typically describing phosphate removal by either first- or second-order expressions derived from Langmuir kinetics^16^, the Elovich model^17^,^18^,^19^, or the intra-particle diffusion model^20^,^21^. While founded in mechanistic principles, the expressions are typically empirical in nature, with little attempt made to relate to particular processes or chemical reactions. Only a few researchers have attempted a more process oriented approach. Shen and Duvnjak^22^ developed a reversible surface reaction model to describe cupric ion adsorption onto corncob particles, in which the kinetic constants for the forward reaction (adsorption) and backward reaction (desorption) were determined. As the protonation of surface sites was neglected in this model, the rate constant for the forward reaction may be overestimated, or, alternatively, the rate constant of the back reaction might be underestimated. In our previous study^7^, although the concentration of H^+^ was extremely low compared to the phosphate concentration, the protonated surface sites were found to dominate over the pH range 6.0–8.0. As such, it can be concluded that the protonation of surface sites competed effectively with the reaction between phosphate and surface sites and, as such, this reaction should be included in any mechanistically-oriented modeling approach.

The rate at which phosphate is removed by Fe(III) is critical in determining the fate, forms and bioavailability of phosphorus in natural water or wastewater at circumneutral pH. In this study, two distinct aqueous systems were used to investigate the kinetics of phosphate removal by iron. First, we investigated the kinetics of phosphate adsorption by preformed HFO (PF-HFO) at circumneutral pH 6.0–8.0, in which millimolar lever of Fe(III) was added to buffer solution to initially form PF-HFO, followed by the subsequent addition of phosphate to the PF-HFO dispersion. Subsequently, we analyzed the kinetics of *in situ* phosphate removal by adding Fe(III) directly into phosphate solutions in the form of a ferric salt. In this case, phosphate was removed from solution by adsorption to *in situ* formed hydrous ferric oxide (ISF-HFO).

The data sets obtained in the two types of studies outlined above were then described using traditional empirical models including the pseudo-second-order model^23^, the Elovich model^17^,^18^,^19^, and the intra-particle diffusion model^20^,^21^. Additionally, the ability of the more mechanistically-based surface complexation kinetic models (SCKMs) for the description of phosphate removal by both PF-HFO and ISF-HFO were investigated.

## Results and Discussion

### Phosphate removal efficiency under various conditions

The rate and extent of phosphate removal were investigated as a function of both initial phosphate concentration and the solution pH. The various conditions under which phosphate removal was examined are shown in Supplementary Table S1.

In the PF-HFO adsorption process, relatively rapid phosphate adsorption was observed in the initial 60 min followed by slower phosphate uptake. The higher the initial phosphate concentration used, the larger the absolute amount but the smaller the proportion of phosphate was adsorbed by 1 mM PF-HFO. The extent of adsorption was due to the relatively high availability of sites on the PF-HFO surface for phosphate adsorption when a higher solute concentration gradient was used. An increase of pH was found to decrease the phosphate adsorption efficiency by PF-HFO dramatically, due to the change in the nature of the surface species involved in phosphate adsorption. This effect has been investigated previously and is accounted for by the molecular models used to describe phosphate adsorption in that study^7^.

At each condition investigated in this study, the efficiency of phosphate removal using Fe(III) added directly into phosphate solutions (*in situ* process) was significantly higher than that when the same concentration of phosphate was added to the PF-HFO suspension formed by same amount of iron. Since the production of strengite (FePO_4_(s)) to achieve high phosphate removal efficiencies has been shown to be extremely unlikely if Fe(III) salts are used, the *in situ* phosphate removal process can be considered an alternative adsorption process to that of phosphate adsorption to PF-HFO. In the process of phosphate adsorption onto PF-HFO, much of the HFO would form in the absence of phosphate, making internal sites unavailable for binding^6^. However, the *in situ* formed solid with an open structure^6^ provides the advantage of a greater number of surface sites than that in the case for preformed HFO. In addition to the greater phosphate uptake capacity, the rate of phosphate removal in the *in situ* process was much higher than that in the PF-HFO case, with most of the phosphate removal being completed in the first 30 min. In a manner similar to that of the PF-HFO adsorption process, the amount of phosphate removed by a particular concentration of Fe(III) in the *in situ* removal process increased with increasing initial phosphate concentration. Unlike the PF-HFO adsorption process, however, the phosphate removal efficiency was not sensitive to changes in solution pH. The reason for this very distinct difference will be discussed below.

### Traditional kinetic models of phosphate adsorption by preformed and *in situ* formed hydrous ferric oxide

To evaluate the effects of the initial phosphate concentration and pH on phosphate adsorption rates, a pseudo-second-order model was used to describe the kinetics of phosphate adsorption by PF-HFO.

Supplementary Fig. S1 demonstrates that a pseudo-second-order model provides a good description of the results obtained for the different initial phosphate concentrations (0.25 mM, 0.5 mM and 1 mM) and pH values (6.0, 7.0, and 8.0) in our study. The corresponding model parameters calculated from the intercepts and slopes of these lines, including *q*_*e*_, *k*_*sec*_, and *h*, are summarized in Supplementary Table S2. From this table, the effect of initial phosphate concentration and pH on the phosphate adsorption efficiency is quantified using *q*_*e*_, which increases with increasing initial phosphate concentration and decreasing pH. Others have concluded^24^,^25^ that phosphate adsorption can be described by the physical diffusion of phosphate in solution, in the liquid film surrounding particles and within the pores of the PF-HFO particles, followed by chemical reaction between the phosphate species and the PF-HFO surface sites. As such, the results determined here for the effect of pH on the rate constants suggests that the initial adsorption rate was determined, for the most part, by fast chemical reactions between phosphate and surface sites, while the rates at later times were most likely determined by physical diffusion effects.

In the *in situ* phosphate removal process, phosphate may either interact with added ferric cations and form distinct assemblages of strengite (FePO_4_(s)), or it may be adsorbed by *in situ* formed HFO. We have noted earlier that the conditions in wastewater treatment are not particularly suited to strengite formation and, as such, phosphate adsorption is the most likely mechanism of phosphate removal. Therefore, to evaluate the rate of *in situ* phosphate removal, the pseudo-second-order model was also used to describe the kinetics of phosphate removal. The model results are shown in Supplementary Fig. S2, and the model parameters derived from the model fits are presented in Supplementary Table S3. As with the PF-HFO adsorption process, the application of the pseudo-second-order model to the description of the *in situ* data is successful. However, the parameters are significantly different to those obtained for the PF-HFO adsorption process. It is obvious that phosphate removal via the *in situ* process is much faster than that for the PF-HFO adsorption process for any particular condition, resulting in higher phosphate removal efficiencies. An increase in the initial concentration of phosphate significantly reduces the phosphate removal rates (*k*_*sec*_), but a change in pH does not significantly impact rates of phosphate removal (*k*_*sec*_). The decrease of *k*_*sec*_ with increasing initial concentration of phosphate is a commonly known fact (the higher the initial concentration, the longer time is required to reach equilibrium). However, the influence of pH on the value of *k*_*sec*_ has not been theoretically investigated, which may be due to its complexity. It is suspected that the phosphate removal rates via an *in situ* process in this study are principally dependent on the physical factors associated with the configuration rather than the chemical reactions between phosphate and the sorbing phase.

The Elovich equation was also used to model the removal of phosphate by PF-HFO with reasonable linear relationships between ln *t* and *q*_*t*_ being obtained for the various sets of phosphate removal data (Supplementary Fig. S3). The Elovich coefficients calculated from the intercepts and slopes of these straight lines are shown in Supplementary Table S3. Generally, the initial adsorption rate *A* decreased and the desorption constant *B* increased with increasing pH (except for the unusually low *B* value in expt no. 6) and both *A* and *B* decreased as the initial phosphate concentration increased (except for the unusual *A* values in expt nos 5 and 9). Although a variety of explanations have been provided^25^ for the observed changes in the Elovich model parameters with changes in [PO_4_^3−^]_T_ and pH, a definitive mechanism providing a rationalization for these changes has not yet been presented. Plazinski *et al*.^24^ concluded that both the Elovich model and the pseudo-second-order model provide similar descriptions of phosphate adsorption kinetics and are special forms of the “generalized-Elovich” model proposed by Rudzinski and Plazinski^26^.

The Elovich equation was unsuccessful in describing the kinetics of *in situ* phosphate removal as indicated by the poor fit of *q*_*t*_ against t in Supplementary Fig. S4. Because the Elovich equation neglects desorption, *q*_*t*_ would be expected to continually increase at extended adsorption times. As such, the Elovich equation is clearly inappropriate for description of *in situ* phosphate removal process where equilibrium is nearly attained.

The intra-particle diffusion model was applied in order to assess the importance of intra-particle diffusion in the kinetics of phosphate adsorption by PF-HFO and *in situ* phosphate removal. As found for the Elovich model, the intra-particle diffusion model was only suitable for describing phosphate adsorption by PF-HFO with reasonable fits of Eq. (7) to the phosphate adsorption data, especially at higher initial phosphate concentrations (Supplementary Fig. S5). The values of the intra-particle diffusion rate constant, *k*_*int*_, obtained by plotting *q*_*t*_ versus *t*_*1/2*_ for various phosphate concentrations and pH values, are shown in Supplementary Table S4. Further, by assuming that PF-HFO assemblages consist of spherical particles, the diffusion coefficients for the intra-particle transport of aqueous phosphate species within the PF-HFO pores were calculated using Eq. (1)^27^ and Eq. (2)^28^,


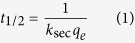



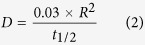


where *D* (cm^2^ min^−1^) is the diffusion coefficient, *R* (cm) represents the average radius of PF-HFO (and is equal to 6.26 × 10^−4^ cm which was calculated using the data in our previous study^7^), and *t*_*1/2*_ (min) is the half time of phosphate adsorption.

As shown in Supplementary Fig. S5, none of the plots passed through the origin, indicating that intra-particle diffusion was not the rate-limiting step for phosphate adsorption by PF-HFO^20^,^29^. Also, the correlation coefficients (*r*^2^) of the fits varied significantly with the goodness of the fits improving as the initial phosphate concentration increased. These results suggest that the intra-particle diffusion effects were more important at higher initial phosphate concentrations, with the intra-particle diffusion rate constants, *k*_*int*_, generally increasing with increasing initial phosphate concentrations.

In addition, as shown in Supplementary Table S4, although the diffusion coefficient *D* increased, the half time of phosphate adsorption, *t*_*1/2*_, had the opposite trend and decreased as the initial phosphate concentration and pH decreased. Both *D* and *t*_*1/2*_ were calculated using the parameters from the pseudo-second-order model, with *D* being found to be on the order of 10^−8^ cm^2^ min^−1^. As such, intra-particle phosphate diffusion is not important in the early stage of phosphate adsorption, although it would play an important role in the later stages of phosphate adsorption^30^,^31^ and could result in the time for phosphate removal varying for several days (as indeed has been previously observed^7^).

Data obtained for *in situ* phosphate removal were poorly described by the intra-particle diffusion model (Fig. S6).

### Surface complexation kinetic models for phosphate adsorption by PF-HFO

According to the rate expressions derived earlier for the surface complexation reactions, while the rate constants should vary with pH, a correlation between the initial concentration of phosphate and the rate constants is not expected. As such, we initially modeled the data sets at different initial concentrations of phosphate using only one set of rate constants in both the SCKM-1 and SCKM-2 models. The results of these initial analyses are shown in Supplementary Fig. S7 and the model parameters are presented in Table 1. Perhaps not surprisingly, given the larger number of fitting parameters, the SCKM-2 model is seen to provide a better fit to the data than the SCKM-1 model. The desorption rate constants *k*_*2*_ derived from the SCKM-2 fitting were extremely low at every pH (6.0–8.0) and, as such, are not shown in Table 1. The fact that phosphate desorption is insignificant means that the SCKM-2 model is, in essence, equivalent to the SCKM-3 model under the conditions used in this study. From Table 1, the adsorption rate constant *k*_*1*_*′* derived from the SCKM-2 model (0.0680–0.274 mM^−1^ min^−1^) is higher than the same constant derived using the SCKM-1 model (0.0386–0.205 mM^−1^ min^−1^). This means that neglecting the protonation of surface sites led to an underestimation of the adsorption rate constant. It is also noteworthy that *k*_*1*_*′* decreased with increasing pH for both the SCKM-1 and SCKM-2 models while the protonation reaction rate constant *k*_*a*_*′* in SCKM-2 (0.0776–0.0947 mM^−1^ min^−1^) increased over the pH range 6.0–8.0. This result suggests that the deprotonation reaction is an important determinant of phosphate adsorption to PF-HFO.

With regard to the influence of initial phosphate concentration on the phosphate adsorption kinetics, the three models were fit to the various data sets available. The solid and dotted lines in Supplementary Fig. S8 represent the results of the application of SCKM-1 and SCKM-2 models while the dotted lines in Supplementary Fig. S9 represent the results of SCKM-3 modeling. It is clear from these results that both the SCKM-2 and SCKM-3 models describe the phosphate adsorption onto PF-HFO quite well, while SCKM-1, with a much shorter equilibrium time, demonstrates larger errors. The parameter values obtained for the different models are shown in Table 2, with both the values for the phosphate adsorption rate constants, *k*_*a*_*′*, for the SCKM-2 and SCKM-3 models evident with the values being higher than the constant determined using the SCKM-1 model.

Since the SCKM-2 and SCKM-3 models provide a better description of phosphate adsorption than the SCKM-1 model, further analysis of the results obtained through use of the SCKM-2 and SCKM-3 models is provided below.

It is obvious that the adsorption rate constant *k*_*a*_*′* decreases dramatically with increasing in pH. To quantify this dependency further, the function 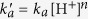 can be transformed as:


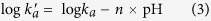


**Figure 1:**
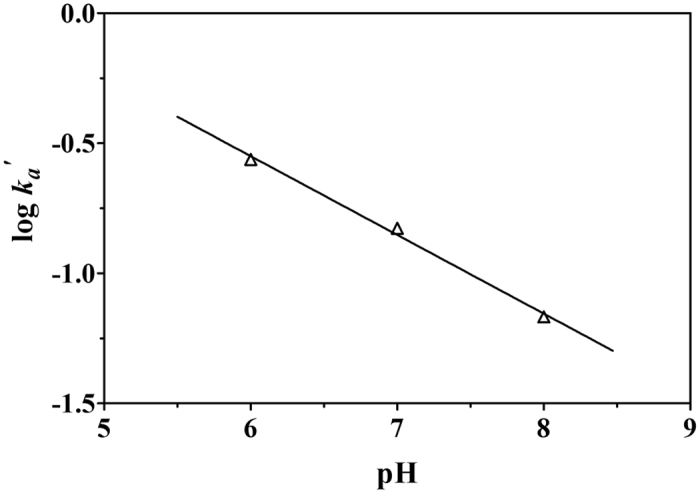
Linear dependence of log *k*_*a*_*′* values on pH determined from SCKM-2 fitting.

Thus a plot of the log *k*_*a*_*′* versus pH should be a straight line with a slope of −*n* and a y-intercept of the log *k*_*a*_ (the intrinsic adsorption rate constant). Linear fits to the model parameters obtained using the SCKM-2 model (Table 1) are shown in Fig. 1. The log *k*_*a*_ and *n* values obtained are 1.266 and 0.302, respectively, with *r*^2^ = 0.982. Linear fitting data for the model parameters obtained using SCKM-3 (Table 2) are given in Table 3 (and the fitting figures are shown in Supplementary Fig. S10), from which a range of log *k*_*a*_ from 0.787 to 1.420, with an average value 1.06 ± 0.33 and a range of n of 0.212–0.316, with an average value 0.255 ± 0.054 were obtained. In general, the *r*^2^ values are above 0.84, indicating good agreement between our model and the observed data. Thus, the adsorption rate constant for phosphate adsorbed onto PF-HFO can be deduced at other pH values, using Eq. 3 with constants log *k*_*a*_ = 1.06 ± 0.33 and n = 0.255 ± 0.054.

According to Hiemstra *et al*.^32^, the point of zero charge can be simplified as log *K*_*H*_ = PZC. Thus,


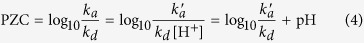


Once *k*_*a*_*′* and *k*_*d*_ have been estimated using the surface complexation kinetic models, PZC can be calculated using Eq. (4). The results deduced by this approach shown in Tables 1 and 2. The range of PZC values obtained is 6.19–9.91, with an average value 8.23 ± 1.02, which is close to the value for HFO determined by Hiemstra and Riemsdijk (PZC = 8.1)^33^.

**Figure 2:**
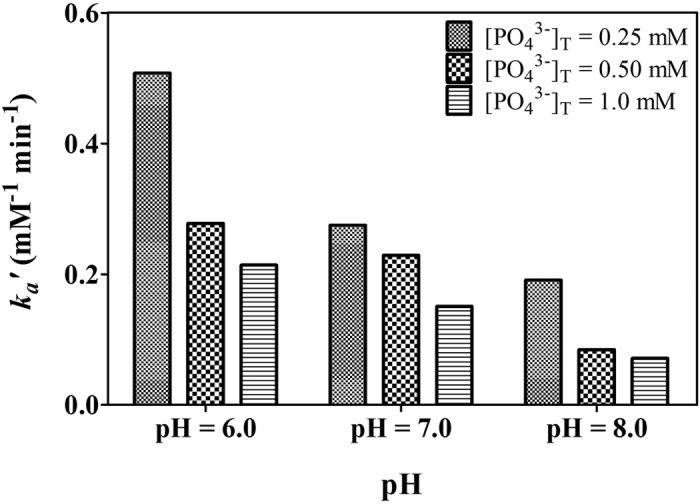
Values of phosphate adsorption rate constant *k*_*a*_ for particular initial phosphate concentrations and pH.

In the surface complexation kinetic models, the phosphate adsorption rate constant *k*_*a*_*′* was observed to decrease with increasing initial phosphate concentration (Fig. 2). A possible reason for this relationship could be the neglect of physical diffusion. Although the experimental conditions in the studies described here including temperature and stirring rate, have been constantly maintained, the initial concentration of phosphate would influence the diffusion rate and, as a result, influence the phosphate adsorption rate.

### Surface complexation kinetic models for *in situ* phosphate removal by Fe(III)

**Figure 3:**
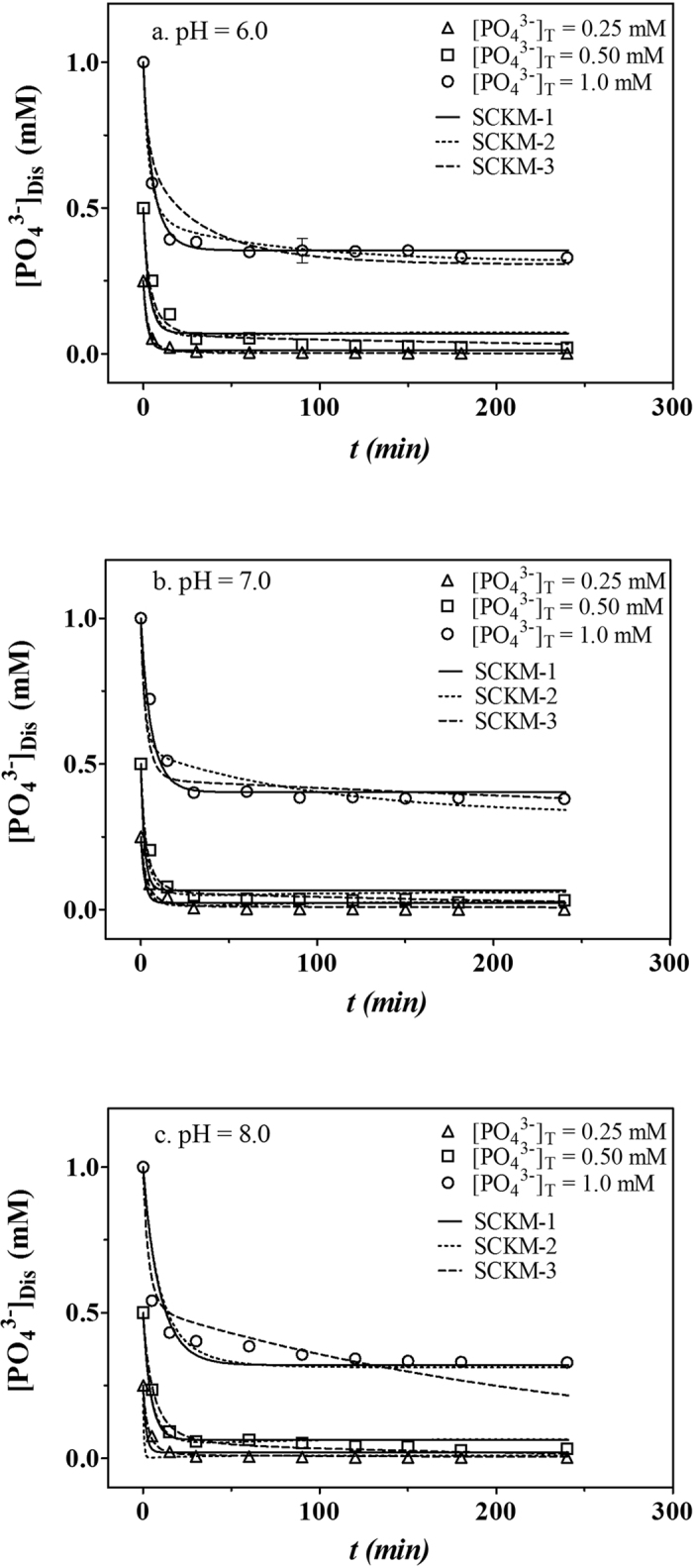
SCKM-1 (solid lines), SCKM-2 (dotted lines), and SCKM-3 (dashed lines) fits for 0.25 mM (△), 0.5 mM (◻), and 1.0 mM (⚪) phosphate *in situ* removal by 1 mM Fe(III) at *I* = 0.1 M NaCl and pH = 6.0 (**a**), 7.0 (**b**), and 8.0 (**c**). Error bars are the standard error of the mean from triplicate experiments.

As mentioned above, the reason for the greater extent of phosphate removal by *in situ* formed hydrous ferric oxide (compared to preformed hydrous ferric oxide) is the greater number of active surface sites on the *in situ* formed solid than on the preformed HFO (the concentration of active surface sites (*Css*) was 0.45 mM (mM Fe)^−1^ in our previous study^7^). To quantify the amount of active surface sites on the *in situ* formed solid, we used the surface complexation kinetic models, with the parameters presented in Table 2, to evaluate the variation of *Css*. The results of these calculations are presented in Table 2. The fits using SCKM-1, SCKM-2, and SCKM-3 for *in situ* phosphate removal by Fe(III) are shown in Fig. 3. It was observed that all three of the surface complexation models could describe *in situ* phosphate removal by Fe(III) quite well. As shown in Table 2, the average values of *Css* for pH = 6.0, 7.0 and 8.0 are 0.955 ± 0.170, 1.46 ± 0.39, and 2.98 ± 0.78 mM (mM Fe)^−1^, respectively, which are larger than the value of 0.45 mM (mM Fe)^−1^ obtained for PF-HFO. However, as initial phosphate concentration at pH 6.0–8.0 was increased, the values of *Css* did not increase accordingly. The rate of HFO polymer formation will be much faster than that of phosphate adsorption by HFO. Hence, it is not surprising that a slight increase in phosphate concentration had little impact on the surface site concentration. In solution, when the iron atom was present as hydrated Fe(H_2_O)_6_^3+^, the value of *Css* would reach its limits of 6 mM ([Fe], mM)^−1^, and for solid HFO, the in air value of *Css* would be 3 mM ([Fe], mM)^−1^ according to stoichiometric composition.However, for a 1-nm cube particle of HFO in solution, the value of *Css* was calculated to be 4.5 mM ([Fe], mM)^−1 6^. In this study, the values of *Css* for *in situ* formed HFO increased with increasing pH and reached 2.98 ± 0.78 mM ([Fe], mM)^−1^ at pH = 8.0, which is close to the value determined by Smith *et al*.^6^ for 2-nm cube particles.

A general conclusion of this study is that the kinetics of interaction between phosphate and Fe(III) in solution was not adequately described by traditional kinetic expressions (e.g. the pseudo-second order and the Elovich equations). However, they could be adequately described by models incorporating the acid-base chemistry of surface sites, proton-phosphate competition for surface sites and phosphate surface complexation. The more molecular-based kinetics models (SCKMs) investigated in this study can be promising to understand the phosphate removal by Fe(III), considering that it is a challenge to elucidate the realistic system by the traditional empirical models. Additionally, the rate of phosphate removal by Fe(III) was affected by the manner of adding Fe(III). This can be achieved in phosphate wastewater treatment by ensuring that iron salts are added *in situ* into the wastewater such that more active surface sites on HFO are produced to obtain a higher phosphate removal rate and efficiency. However, the kinetic models and rate constants developed in our study were investigated in the simulated waters containing simple inorganic matrices without the addition of organic compounds. In natural water or wastewater, various organic compounds could complex both Fe(III), and the formed Fe species, which would also affect the formation of HFO and further affect the kinetics of phosphate removal. These effects would need further investigation before the application of the developed models in natural water or wastewater.

## Methods

Analytical grade reagents were used in all cases and purchased from Sigma-Aldrich unless otherwise stated. All solutions were prepared using 18 MΩ cm ultrapure Milli-Q (MQ) water and stored in the dark at 4 °C when not in use. All glassware and plasticware were soaked in 5% v/v HCl for several days and rinsed thoroughly with MQ water before use. All experiments were performed under conditions in which light was minimized to avoid Fe(III) reduction by wrapping the reaction vessel with dark cloth at 22 ± 1 °C.

The pH of all solutions used was carefully controlled with an appropriate buffer (10 mM MES (SigmaUltra) for pH 6.0–6.5 and 10 mM MOPS (SigmaUltra) for pH 7.0–8.0). Both MES and MOPS are non-complexing agents^34^ and are unlikely to influence the behavior of other solution constituents^7^. All buffer solutions initially contained 2.0 mM NaHCO_3_ and 100 mM NaCl and were prepared one week prior to the commencement of each experiment in order to ensure that equilibrium was reached between the buffer solutions and the atmosphere. The chosen buffers maintained solution pH within ± 0.02 pH units of the desired value during the course of each experiment. pH was measured using a HANNA 211 pH meter combined with a glass electrode and Ag/AgCl reference. NIST-traceable buffer solutions (pH 4.01, 7.01 and 10.01) were used to calibrate the electrode on the NBS scale. Any pH error due to ionic strength differences between the NIST standard buffers (pH 4.01, *I* = 0.05; pH 7.01, *I* = 0.13; and pH 10.01, *I* = 0.05) and experimental solutions (*I* ~ 0.1 in this work) is minor and can be neglected.

A 100 mM Fe(III) stock solution was prepared by dissolving an appropriate amount of FeCl_3_ in 2 mM HCl. A 1.0 M phosphate stock solution was prepared by dissolving an appropriate amount of NaH_2_PO_4_ in MQ water. PF-HFO were prepared by adding 1 mM Fe(III) into the particular buffer solution (pH 6.0–8.0) with stirring at 25 rpm and pH readjusted to the specific value required. Speciation calculations using the Visual Minteq 3.0 chemical equilibrium program^35^ indicated that essentially all Fe(III) would be precipitated as HFO at the various pH examined.

### Kinetics of phosphate adsorption by PF-HFO

To determine the rate of phosphate adsorbed by PF-HFO at different pH values, 5-min-aged pre-polymerized HFO containing 1 mM Fe(III) was used as the PF-HFO. The desired phosphate concentration was mixed with 200 mL of 1 mM Fe(III)-containing PF-HFO in 500 mL conical flasks which were agitated on a shaker at a constant speed (120 rpm). Samples were withdrawn at desired intervals and immediately filtered through 25 mM diameter Millex 0.45 μm pore size membrane filters. Adsorption of soluble phosphate on the filter membrane was found to be negligible. The dissolved phosphate concentration ([PO_4_^3−^]_Dis_) was measured using the ascorbic acid molybdate blue method, and the colored complex was detected using a Cary 50 UV-Vis spectrophotometer. The concentration of removed phosphate ([PO_4_^3−^]_ads_) is equal to the initial phosphate concentration ([PO_4_^3−^]_T_) minus the dissolved phosphate concentration. Experiments were repeated in triplicate for the different initial phosphate concentrations (0.25 mM, 0.5 mM and 1 mM) and pH values (6.0, 7.0 and 8.0).

### Kinetics of *in situ* phosphate removal

To determine the phosphate removal capacity and rate for the *in situ* process, the desired concentrations of phosphate were first added to 200 mL buffer solutions in 500 mL conical flasks, and then Fe(III) was added from the 100 mM stock solution such that the reaction mixture had an Fe(III) concentration of 1 mM. The flasks were agitated on a shaker at 180 rpm for the initial 5 min and then at 120 rpm for the remaining time. The approach to sample treatment and phosphate measurement was identical to that used in the investigation of PF-HFO described above. Experiments were repeated in triplicate for the different initial phosphate concentrations (0.25 mM, 0.5 mM, and 1 mM) and pH values (6.0, 7.0, and 8.0).

### Traditional kinetic models of phosphate removal

The removal efficiencies (*E*, *%*) of phosphate and the sorption capacity (*q*, ([P], mM)·([Fe], mM)^−1^) in the *in situ* process and the PF-HFO adsorption process were calculated from Eqs. (5) and (6):


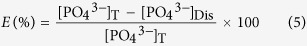



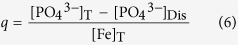


where [Fe]_T_ (in mM of Fe) denotes the total concentration of HFO in solution.

When treating phosphate adsorption as a second order process, the relationship of sorption capacity (*q*_*t*_, ([P], mM) ([Fe], mM)^−1^) and adsorption time (*t*, min) can be linearly expressed as^23^:


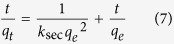


where *k*_*sec*_ (([Fe], mM) ([P], mM)^−1^ min^−1^) represents the second-order rate constant for phosphate adsorption and *q*_*e*_ (([P], mM) ([Fe], mM)^−1^) is the sorption capacity at adsorption equilibrium. The initial adsorption rate *h* (([P], mM) ([Fe], mM)^−1^ min^−1^) may be calculated using the following equation^23^:


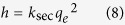


The Elovich model proposed by Roginsky and Zeldovich^17^ was also adopted to describe the kinetics of phosphate adsorption. The integral form of this equation can be depicted as^18^,^19^:


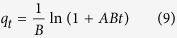


where *A* (([P], mM) ([Fe], mM)^−1^ min^−1^) is the initial sorption rate and *B* (([Fe], mM) ([P], mM)^−1^) is the desorption constant. When *ABt* ≫ 1, this equation can be linearized to:


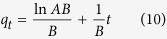


Recognizing that the kinetics of phosphate adsorption may be controlled by diffusion processes, Weber and Morris^20^ and McKay and Poots^21^ introduced the intra-particle diffusion model:


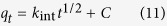


where *C* (([P], mM) ([Fe], mM)^−1^) is the intercept and *k*_*int*_ (([P], mM) ([Fe], mM)^−1^ min^−1/2^) is the intra-particle diffusion rate constant.

### One reversible reaction modeling process (SCKM-1)

According to Langmuir^36^, adsorption can be regarded as a reversible process between the adsorbent and adsorbate. In our previous study, the adsorption of phosphate onto PF-HFO was conceptualized as a “surface complexation” reaction^7^:


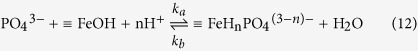


where *k*_*a*_ and *k*_*d*_ represent the adsorption and desorption rate constants, PO_4_^3−^ represents the various possible solution-phase phosphate species (which are assumed to interchange rapidly), ≡FeOH represents the active surface site on PF-HFO, which was determined to be 0.45 mM ([Fe], mM)^−1^ in our previous study^7^ (though protonated or deprotonated forms may dominate depending upon the pH), and ≡FeH_n_PO_4_^(3−n)−^ was considered to be representative of the phosphate “surface complex”, which forms on the surface of HFO (and which is simplified to ≡FeP below). It should be noted that the phosphate surface complex used in this study was semi-empirical and that the actual surface speciation of HFO was complicated and should be further investigated by ATR-FTIR and XAFS^37^.

By applying the mass action law to the reaction, the overall rate equation for adsorption can be expressed as follows:


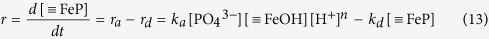


where *r*, *r*_*a*_ and *r*_*d*_ represent the overall rate, the adsorption rate and the desorption rate, respectively and *n* represents the number of protons associated with each “surface complex”.

If the pH is constant during phosphate sorption, the overall rate equation may be simplified to:


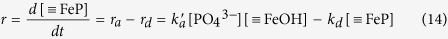


where 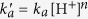. When the initial concentration of phosphate, concentration of total active surface sites and concentration of occupied surface active sites ≡FeP are represented as *C*_*p*_, *C*_*s*_, and *P*_*ads*_, respectively, the overall rate equation becomes:


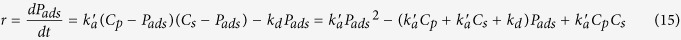


when *P*_*ads*_ = 0 at *t* = 0, Eq. (11) yields:


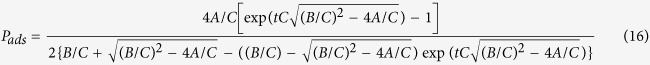


where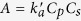, 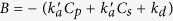, and 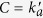. According to Eq. (16), *k*_*a*_*′* and *k*_*d*_ can be obtained using the least square method.

### Two reversible reactions modeling process (SCKM-2)

We have previously shown that the protonation of surface sites occurs in parallel with the adsorption of phosphate^7^. Hence, surface site protonation:


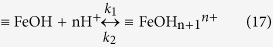


where *k*_*1*_ and *k*_*2*_ represent the protonation and deprotonation rate constants, and ≡FeOH_n+1_^n+^ represents the protonated surface sites (shortened to ≡FeOH^+^ below) should also be considered when modeling phosphate adsorption.

If the pH is constant during phosphate sorption, the pH dependence of the process can be incorporated in the rate expression by use of the rate constant:


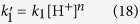


When *k*_*1*_*′*, *k*_*2*_, *k*_*a*_*′*, and *k*_*d*_ are all treated as unknown parameters requiring evaluation, the differential equations describing the kinetics of phosphate adsorption are:


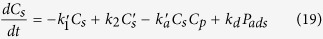



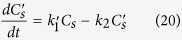



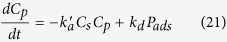



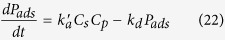


where *C*_*s*_*′* represents the concentration of protonated surface sites present. As these differential equations cannot be solved analytically, we have used the numerical methods package in DynaFit^38^ to model the time dependent phosphate adsorption process in this study.

### One reversible reaction and one irreversible reaction modeling process (SCKM-3)

In our previous study^7^, we observed that the equilibrium constants for phosphate adsorption to PF-HFO were extremely high, which means that the adsorption rate is much higher than the desorption rate. As such, Eq. (8) can be rewritten as:


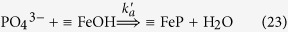


and the differential equations describing the phosphate adsorption process simplified to:


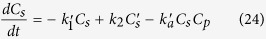



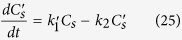



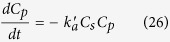



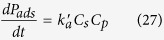


The DynaFit approach was again used to model the experimental data on phosphate adsorption kinetics obtained and to derive values for the parameters *k*_*1*_*′*, *k*_*2*_, and *k*_*a*_*′*.

## Additional Information

**How to cite this article**: Mao, Y. and Yue, Q. Kinetic Modeling of Phosphate Adsorption by Preformed and In situ formed Hydrous Ferric Oxides at Circumneutral pH. *Sci. Rep.* **6**, 35292; doi: 10.1038/srep35292 (2016).

## Supplementary Material

Supplementary Information

## Figures and Tables

**Table 1:** Model parameters for SCKM-1 and SCKM-2 at different pH values^a^.

Model parameters	pH = 6		pH = 7		pH = 8	
	SCKM-1	SCKM-2	SCKM-1	SCKM-2	SCKM-1	SCKM-2
*k*_*1*_*′*(min^−1^)	—	0.0776	—	0.0856	—	0.0947
*k*_*2*_ (min^−1^)	—	0.00329	—	0.00239	—	0.00381
*k*_*a*_*′* (mM^−1^ min^−1^)	0.205	0.274	0.129	0.149	0.0386	0.0680
*k*_*d*_ (min^−1^)	0.0279	—	0.0470	—	0.0309	—
PZC	—	7.37	—	8.55	—	9.39

^a^The model parameters were obtained when the influence of initial phosphate concentration was neglected.

**Table 2:** Model parameters for SCKM-1, SCKM-2, and SCKM-3 at different pH values and different initial phosphate concentrations^a^.

	[PO_4_^3−^]_T_ = 0.25 mM			[PO_4_^3−^]_T_ = 0.50 mM			[PO_4_^3−^]_T_ = 1.0 mM		
	SCKM-1	SCKM-2	SCKM-3	SCKM-1	SCKM-2	SCKM-3	SCKM-1	SCKM-2	SCKM-3
pH=6									
*k*_*1*_*′*(min^−1^)	—	0.0674	0.0657	—	0.0683	0.0675	—	0.0678	0.119
*k*_*2*_ (min^−1^)	—	0.0122	0.000604	—	0.0112	0.00148	—	0.0168	0.0776
*k*_*a*_*′* (mM^−1^ min^−1^)	0.433	0.519	0.508	0.242	0.285	0.278	0.134	0.235	0.214
*k*_*d*_(min^−1^)	0.0177	0.0029	—	0.0323	0.00457	—	0.029	0.00264	—
*C*_*ss*_ (mM mM)^−1^^ b^	0.45	0.45	0.45	0.45	0.45	0.45	0.45	0.45	0.45
*C*_*ss*_ (mM mM)^−1^^ c^	0.919	1.03	0.807	1.25	1.09	0.958	1.04	0.809	0.694
PZC	—	6.74	8.04		6.79	7.66		6.61	6.19
pH=7									
*k*_*1*_*′*(min^−1^)	—	0.0788	0.0802	—	0.108	0.106	—	0.219	0.176
*k*_*2*_ (min^−1^)	—	0.00489	0.000485	—	0.00403	0.00166	—	0.0137	0.00614
*k*_*a*_*′* (mM^−1^ min^−1^)	0.241	0.276	0.275	0.192	0.232	0.229	0.0779	0.170	0.151
*k*_*d*_ (min^−1^)	0.0382	0.00223	—	0.0552	0.00138	—	0.0432	0.00288	—
*C*_*ss*_ (mM mM)^−1^^ b^	0.45	0.45	0.45	0.45	0.45	0.45	0.45	0.45	0.45
*C*_*ss*_ (mM mM)^−1 ^c	1.72	1.33	1.14	2.31	1.59	1.44	1.42	1.28	0.931
PZC		8.21	9.22		8.43	8.81		8.20	8.46
pH=8									
*k*_*1*_*′*(min^−1^)	—	0.0598	0.0984	—	0.0677	0.0579	—	0.0398	0.195
*k*_*2*_ (min^−1^)	—	0.0168	0.00121	—	0.0127	0.0026	—	0.0256	0.00671
*k*_*a*_*′* (mM^−1^ min^−1^)	0.152	0.214	0.191	0.0499	0.0705	0.0847	0.0227	0.0255	0.0715
*k*_*d*_(min^−1^)	0.0470	0.0175	—	0.0275	0.00462	—	0.0279	0.0108	—
*C*_*ss*_ (mM mM)^−1^^ b^	0.45	0.45	0.45	0.45	0.45	0.45	0.45	0.45	0.45
*C*_*ss*_ (mM mM)^−1^ c	3.68	3.06	1.85	4.26	3.24	2.03	3.29	3.07	2.37
PZC		8.55	9.91		8.73	9.35		8.19	9.46

^a^The model parameters were obtained when the influence of initial phosphate concentration was considered.

^b^The active surface sites^7^ were used for PF-HFO case.

^c^The active surface sites for ISF-HFO case were determined by using the rate constants obtained in the PF-HFO case.

**Table 3:** Linear fitting data of adsorption constants in SCKM-3.

	[PO_4_^3−^]_T_ = 0.25 mM	[PO_4_^3−^]_T_ = 0.50 mM	[PO_4_^3−^]_T_ = 1.0 mM	Ave (mM)
log *k*_*a*_	0.962	1.420	0.787	1.06 ± 0.327
*n*	0.212	0.316	0.238	0.255 ± 0.054
*r*^2^	0.978	0.847	0.957	—

## References

[b1] Fytianos K., Voudrias E. & Raikos N. Modelling of phosphorus removal from aqueous and wastewater samples using ferric iron. Environ. Pollut. 101, 123–130 (1998). 15093105 10.1016/s0269-7491(98)00007-4

[b2] Ferreira S. S., Marguti A. L. & Piveli R. P. Physical-Chemical Process Optimization for Phosphorus Removal from Domestic Wastewater by Chemical Precipitation with Ferric Chloride. Eng. Sanit. Ambient. 13, 395–404 (2008).

[b3] Sisk L., Benefield L. & Reed B. Ortho-Phosphate Removal from a Synthetic Waste-Water Using Lime, Alum, and Ferric-Chloride. Separ. Sci. Technol. 22, 1471–1501 (1987).

[b4] Thistleton J., Berry T. A., Pearce P. & Parsons S. A. Mechanisms of chemical phosphorus removal II - Iron(III) salts. Process Saf. Environ. 80, 265–269 (2002).

[b5] Galarneau E. & Gehr R. Phosphorus removal from wastewaters: Experimental and theoretical support for alternative mechanisms. Water Res. 31, 328–338 (1997).

[b6] Smith S., Takacs I., Murthy S., Daigger G. T. & Szabo A. Phosphate complexation model and its implications for chemical phosphorus removal. Water Environ. Res. 80, 428–438 (2008). 18605382

[b7] Mao Y., Ninh Pham A., Xin Y. & David Waite T. Effects of pH, floc age and organic compounds on the removal of phosphate by pre-polymerized hydrous ferric oxides. Sep. Purif. Technol. 91, 38–45 (2012).

[b8] Luengo C., Brigante M. & Avena M. Adsorption kinetics of phosphate and arsenate on goethite. A comparative study. J. Colloid. Interf. Sci. 311, 354–360 (2007). 10.1016/j.jcis.2007.03.027 17448491

[b9] Luengo C., Brigante M., Antelo J. & Avena M. Kinetics of phosphate adsorption on goethite: Comparing batch adsorption and ATR-IR measurements. J. Colloid. Interf. Sci. 300, 511–518 (2006). 10.1016/j.jcis.2006.04.015 16643942

[b10] Mezenner N. Y. & Bensmaili A. Kinetics and thermodynamic study of phosphate adsorption on iron hydroxide-eggshell waste. Chem. Eng. J. 147, 87–96 (2009).

[b11] Shi Z.-l., Liu F.-m. & Yao S.-h. Adsorptive removal of phosphate from aqueous solutions using activated carbon loaded with Fe(III) oxide. New Carbon Mater. 26, 299–306 (2011).

[b12] Song X., Pan Y., Wu Q., Cheng Z. & Ma W. Phosphate removal from aqueous solutions by adsorption using ferric sludge. Desalination 280, 384–390 (2011).

[b13] Zeng L., Li X. & Liu J. Adsorptive removal of phosphate from aqueous solutions using iron oxide tailings. Water Res. 38, 1318–1326 (2004). 14975665 10.1016/j.watres.2003.12.009

[b14] Shang C., Stewart J. W. B. & Huang P. M. pH effect on kinetics of adsorption of organic and inorganic phosphates by short-range ordered aluminum and iron precipitates. Geoderma 53, 1–14 (1992).

[b15] Crosby S. A., Millward G. E., Butler E. I., Turner D. R. & Whitfield M. Kinetics of phosphate adsorption by iron oxyhydroxides in aqueous systems. Estuar. Coast. Shelf Sci. 19, 257–270 (1984).

[b16] Liu Y. & Shen L. From Langmuir Kinetics to First- and Second-Order Rate Equations for Adsorption. Langmuir 24, 11625–11630 (2008). 18788769 10.1021/la801839b

[b17] Roginsky S. Z. & Zeldovich J. Acta Physicochim. USSR 1, 554–594 (1934).

[b18] Taylor H. A. & Thon N. Kinetics of Chemisorption1. J. Am. Chem. Soc. 74, 4169–4173 (1952).

[b19] Low M. J. D. Kinetics of Chemisorption of Gases on Solids. Chem. Rev. 60, 267–312, 10.1021/cr60205a003 (1960).

[b20] Weber W. J. & Morris J. C. Kinetics of adsorption on carbon from solution. J. Sanit. Eng. Div. ASCE 89, 31–59 (1963).

[b21] McKay G. & Poots V. J. P. Kinetics and diffusion processes in colour removal from effluent using wood as an adsorbent. J. Chem. Technol. Biot. 30, 279–292 (1980).

[b22] Shen J. & Duvnjak Z. A reversible surface reaction model with an effectiveness factor and its application to sorption kinetics of cupric ions on corncob particles. Sep. Purif. Technol. 44, 69–77 (2005).

[b23] Ho Y.-S. Review of second-order models for adsorption systems. J. Hazard. Mater. 136, 681–689 (2006). 16460877 10.1016/j.jhazmat.2005.12.043

[b24] Plazinski W., Rudzinski W. & Plazinska A. Theoretical models of sorption kinetics including a surface reaction mechanism: A review. Adv. Colloid Interfac. 152, 2–13 (2009). 10.1016/j.cis.2009.07.009 19735907

[b25] Sen Gupta S. & Bhattacharyya K. G. Kinetics of adsorption of metal ions on inorganic materials: A review. Adv. Colloid Interfac. 162, 39–58 (2011). 10.1016/j.cis.2010.12.004 21272842

[b26] Rudzinski W. & Plazinski W. On the applicability of the pseudo-second order equation to represent the kinetics of adsorption at solid/solution interfaces: a theoretical analysis based on the statistical rate theory. Adsorption 15, 181–192 (2009).

[b27] Rauf M. A., Bukallah S. B., Hamour F. A. & Nasir A. S. Adsorption of dyes from aqueous solutions onto sand and their kinetic behavior. Chem. Eng. J. 137, 238–243 (2008).

[b28] Doğan M., Özdemir Y. & Alkan M. Adsorption kinetics and mechanism of cationic methyl violet and methylene blue dyes onto sepiolite. Dyes Pigments 75, 701–713 (2007).

[b29] Arami M., Limaee N. Y. & Mahmoodi N. M. Evaluation of the adsorption kinetics and equilibrium for the potential removal of acid dyes using a biosorbent. Chem. Eng. J. 139, 2–10 (2008).

[b30] Lijklema L. Interaction of Ortho-Phosphate with Iron(III) and Aluminum Hydroxides. Environ. Sci. Technol. 14, 537–541 (1980).

[b31] Makris K. C., El-Shall H., Harris W. G., O’Connor G. A. & Obreza T. A. Intraparticle phosphorus diffusion in a drinking water treatment residual at room temperature. J. Colloid Interf. Sci. 277, 417–423 (2004). 10.1016/j.jcis.2004.05.001 15341854

[b32] Hiemstra T., Venema P. & Riemsdijk W. H. V. Intrinsic Proton Affinity of Reactive Surface Groups of Metal (Hydr)oxides: The Bond Valence Principle. J. Colloid Interf. Sci. 184, 680–692 (1996). 10.1006/jcis.1996.0666 8978574

[b33] Hiemstra T. & Van Riemsdijk W. H. A surface structural model for ferrihydrite I: Sites related to primary charge, molar mass, and mass density. Geochim. Cosmochim. Ac. 73, 4423–4436 (2009).

[b34] Kandegedara A. & Rorabacher D. B. Noncomplexing tertiary amines as “better” buffers covering the range of pH 3–11. Temperature dependence of their acid dissociation constants. Anal. Chem. 71, 3140–3144 (1999). 21662904 10.1021/ac9902594

[b35] Gustafsson J. P. Visual MINTEQ version 3.0. KTH, Stockholm, Sweden. http://vminteq.lwr.kth.se/.

[b36] Langmuir I. The adsorption of gases on plane surfaces of glass, mica and platinum. J. Am. Chem. Soc. 40, 508 (1918).

[b37] Rouff A. A. & Juarez K. M. Zinc Interaction with Struvite During and After Mineral Formation. Environmental Science & Technology 48 (2014). 10.1021/es500188t 24794191

[b38] Kuzmič P. Program DYNAFIT for the analysis of enzyme kinetic data: application to HIV proteinase. Anal. Biochem. 237, 260–273 (1996). 8660575 10.1006/abio.1996.0238

