# Supplementary material for: Kinetic Modeling of Phosphate Adsorption by Preformed and In situ formed Hydrous Ferric Oxides at Circumneutral pH
Source: Sci Rep. 2016 Oct 14;6:35292. doi: 10.1038/srep35292 (PMC5064408; doi:10.1038/srep35292)
Supplement: Supplementary Information [file srep35292-s1.doc]

Supplementary Information for

**Kinetic Modeling of Phosphate Adsorption by Pre-formed and In situ-formed Hydrous Ferric Oxides at Circumneutral pH**

Yanpeng Mao^a^*, Qinyan Yue^b^

^a^ School of Energy and Power Engineering, Shandong University, Jinan 250100, PR China.

^b^ School of Environmental Science and Engineering, Shandong University, Jinan 250100, PR China.

Table S1 The phosphate removal efficiency for PF-HFO adsorption and “in situ” removal on addition of a ferric salt.

| [PO_4_^3–^]_T_ | pH | *E_t_*,% (PF-HFO /in situ) at different time | | | | | |
| --- | --- | --- | --- | --- | --- | --- | --- |
|  |  | 5 min | 15 min | 30 min | 60 min | 120 min | 240 min |
| 0.25 | 6.0 | 51.6±5.0/  79.2±3.5 | 76.1±3.1/  91.2±3.3 | 84.9±6.4/  96.7±0.2 | 84.7±2.4/  98.2±0.6 | 87.2±2.0/  98.7±0.1 | 87.5±2.6/  99.2±0.0 |
|  | 7.0 | 32.3±6.9/  64.4±4.6 | 56.5±2.0/  82.4±2.5 | 60.3±6.3/  97.8±0.4 | 62.0±5.0/  99.0±0.2 | 64.5±4.3/  99.3±0.1 | 64.7±1.7/  99.4±0.0 |
|  | 8.0 | 31.0±4.2/  69.6±6.9 | 38.3±3.0/  91.2±3.8 | 47.7±2.9/  97.2±0.5 | 51.9±4.8/  97.8±0.3 | 53.2±5.2/  98.6±0.1 | 53.5±2.6/  98.7±0.0 |
| 0.5 | 6.0 | 32.2±4.4/  49.8±2.7 | 47.4±3.2/  72.8±1.4 | 53.5±3.6/  89.6±3.3 | 53.8±3.9/  89.4±0.2 | 55.6±2.4/  94.8±0.1 | 58.4±1.0/  95.8±0.1 |
|  | 7.0 | 26.2±5.0/  59.0±2.4 | 37.7±5.0/  84.4±1.2 | 40.4±4.3/  90.4±1.2 | 41.4±2.7/  92.4±1.2 | 43.6±3.3/  93.6±0.4 | 46.8±3.1/  93.6±0.2 |
|  | 8.0 | 11.0±2.3/  53.0±1.2 | 19.5±3.7/  81.8±1.7 | 26.6±3.0/  88.6±1.1 | 29.2±2.6/  87.4±2.3 | 33.2±2.9/  91.8±0.8 | 35.5±0.2/  93.6±0.8 |
| 1.0 | 6.0 | 22.0±3.6/  41.5±1.1 | 26.4±3.2/  60.8±0.7 | 28.4±2.9/  61.7±0.7 | 31.2±2.4/  65.1±1.1 | 34.3±0.9/  64.9±0.4 | 37.3±0.8/  67.0±0.9 |
|  | 7.0 | 16.6±1.5/  27.7±0.9 | 19.1±1.0/  48.9±1.1 | 21.4±0.1/  59.8±1.6 | 23.9±0.2/  59.5±0.3 | 25.6±0.1/  61.4±0.2 | 29.3±0.8/  62.0±0.1 |
|  | 8.0 | 7.7±2.0/  45.9±0.8 | 9.8±2.0/  56.8±0.9 | 11.6±0.9/  59.8±0.6 | 14.1±1.5/  61.5±0.8 | 17.5±1.3/  65.8±0.6 | 20.0±0.7/  67.1±0.3 |

**Table S2 Parameters for Pseudo-second-order model ^a^**

| No. | [PO_4_^3–^]_T_  (mM) | pH | *q_e_* (mM mM^–1^) | | *k_sec_* (mM mM^–1^ min^–1^) | | *h* (mM mM^–1^ min^–1^) | |
| --- | --- | --- | --- | --- | --- | --- | --- | --- |
|  |  |  | Preformed | In situ | Preformed | In situ | Preformed | In situ |
| 1 | 0.25 | 6.0 | 0.221 | 0.251 | 1.99 | 3.48 | 0.0975 | 0.216 |
| 2 | 0.25 | 7.0 | 0.165 | 0.249 | 1.77 | 2.15 | 0.0479 | 0.214 |
| 3 | 0.25 | 8.0 | 0.137 | 0.246 | 1.72 | 3.25 | 0.0322 | 0.201 |
| 4 | 0.5 | 6.0 | 0.296 | 0.486 | 0.702 | 0.574 | 0.0615 | 0.136 |
| 5 | 0.5 | 7.0 | 0.238 | 0.479 | 0.627 | 0.594 | 0.0354 | 0.134 |
| 6 | 0.5 | 8.0 | 0.189 | 0.475 | 0.358 | 0.577 | 0.0127 | 0.131 |
| 7 | 1 | 6.0 | 0.382 | 0.679 | 0.272 | 0.371 | 0.0396 | 0.171 |
| 8 | 1 | 7.0 | 0.302 | 0.681 | 0.266 | 0.412 | 0.0242 | 0.164 |
| 9 | 1 | 8.0 | 0.213 | 0.632 | 0.219 | 0.365 | 0.0100 | 0.170 |

^a^ “Preformed” represents phosphate adsorption by pre-formed HFO; “In situ” represents phosphate removal by in-situ addition of Fe(III) salts

**Table S3** Parameters for Elovich model (phosphate adsorption by preformed HFO)

| No. | [PO_4_^3–^]_T_  (mM) | pH | *A*  (mM mM^–1^ min^–1^) | *B*  (mM mM^–1^) | *r*^2^ |
| --- | --- | --- | --- | --- | --- |
| 1 | 0.25 | 6.0 | 22.0 | 55.4 | 0.796 |
| 2 | 0.25 | 7.0 | 2.26 | 59.6 | 0.773 |
| 3 | 0.25 | 8.0 | 0.627 | 66.3 | 0.913 |
| 4 | 0.5 | 6.0 | 6.51 | 36.4 | 0.848 |
| 5 | 0.5 | 7.0 | 5.47 | 45.9 | 0.890 |
| 6 | 0.5 | 8.0 | 0.0572 | 32.3 | 0.973 |
| 7 | 1 | 6.0 | 2.03 | 25.3 | 0.995 |
| 8 | 1 | 7.0 | 0.543 | 28.6 | 0.976 |
| 9 | 1 | 8.0 | 0.0730 | 31.8 | 0.967 |

**Table S4** Parameters for Intra-particle diffusion model (phosphate adsorption by preformed HFO)

| No. | [PO_4_^3–^]_T_  (mM) | pH | *k_int_* × 10^–3^  (mM mM^–1^ min^–1/2^) | *r*^2^ | *D* × 10^–8^  (cm^2^ min^–1^) | *t_1/2_* |
| --- | --- | --- | --- | --- | --- | --- |
| 1 | 0.25 | 6.0 | 4.35 | 0.623 | 0.514 | 2.27 |
| 2 | 0.25 | 7.0 | 4.06 | 0.655 | 0.341 | 3.42 |
| 3 | 0.25 | 8.0 | 3.86 | 0.735 | 0.275 | 4.24 |
| 4 | 0.5 | 6.0 | 6.90 | 0.656 | 0.243 | 4.81 |
| 5 | 0.5 | 7.0 | 5.63 | 0.726 | 0.174 | 6.70 |
| 6 | 0.5 | 8.0 | 8.25 | 0.849 | 0.079 | 14.8 |
| 7 | 1 | 6.0 | 10.9 | 0.936 | 0.121 | 9.62 |
| 8 | 1 | 7.0 | 9.94 | 0.971 | 0.094 | 12.5 |
| 9 | 1 | 8.0 | 9.02 | 0.978 | 0.054 | 21.4 |

**Figure S1** Pseudo-second-order model fits for 0.25mM (a), 0.5 mM (b) and 1.0 mM (c) phosphate adsorption onto preformed HFO (1 mM Fe) at *I* = 0.1 NaCl and pH = 6.0 (△), 7.0 (□) and 8.0 (○). Error bars are the standard error of the mean from triplicate experiments.

**Figure S2** Pseudo-second-order model fits for 0.25 mM, 0.5 mM and 1.0 mM phosphate “in situ” removal by 1 mM Fe(III) at *I* = 0.1 NaCl and pH = 6.0 (△), 7.0 (□) and 8.0 (○). Error bars are the standard error of the mean from triplicate experiments.

**Figure S3** Elovich model fit for 0.25 mM (a), 0.5 mM (b) and 1.0 mM (c) phosphate adsorption onto preformed HFO (1 mM Fe) at *I* = 0.1 NaCl and pH = 6.0 (△), 7.0 (□) and 8.0 (○). Error bars are the standard error of the mean from triplicate experiments.

**Figure S4** Unsuccessful fitting of Elovich model for 0.25 mM (a), 0.5 mM (b) and 1.0 mM (c) phosphate “in situ” removal by 1 mM Fe(III) at *I* = 0.1 NaCl and pH = 6.0 (△), 7.0 (□) and 8.0 (○). Error bars are the standard error of the mean from triplicate experiments.

**Figure S5** Intra-particle diffusion model fit for 0.25 mM (a), 0.5 mM (b) and 1.0 mM (c) phosphate adsorption onto preformed HFO (1 mM Fe) at *I* = 0.1 NaCl and pH = 6.0 (△), 7.0 (□) and 8.0 (○). Error bars are the standard error of the mean from triplicate experiments.

**Figure S6** Unsuccessful fitting of intra-particle diffusion model for 0.25 mM (a), 0.5 mM (b) and 1.0 mM (c) phosphate “in situ” removal by 1 mM Fe(III) at *I* = 0.1 NaCl and pH = 6.0 (△), 7.0 (□) and 8.0 (○). Error bars are the standard error of the mean from triplicate experiments.

**Figure S7** SCKM-1 (solid lines) and SCKM-2 (dashed lines) fits for 0.25 mM (△), 0.5 mM (□) and 1.0 mM (○) phosphate adsorption onto preformed HFO (1 mM Fe) at *I* = 0.1 NaCl and pH = 6.0 (a), 7.0 (b) and 8.0 (c) when the influence of initial phosphate concentration was neglected. Error bars are the standard error of the mean from triplicate experiments.

**Figure S8** SCKM-1 (solid lines) and SCKM-2 (dashed lines) fits for 0.25 mM (△), 0.5 mM (□) and 1.0 mM (○) phosphate adsorption onto preformed HFO (1 mM Fe) at *I* = 0.1 NaCl and pH = 6.0 (a), 7.0 (b) and 8.0 (c) when the influence of initial phosphate concentration was considered. Error bars are the standard error of the mean from triplicate experiments.

**Figure S9** SCKM-3 fits for 0.25 mM (△), 0.5 mM (□) and 1.0 mM (○) phosphate adsorption onto preformed HFO (1 mM Fe) at *I* = 0.1 NaCl and pH = 6.0 (a), 7.0 (b) and 8.0 (c) when the influence of initial phosphate concentration was considered. Error bars are the standard error of the mean from triplicate experiments.

**Figure S10 Linear fittings for the model parameters obtained using SCKM-3 in Table 5.**
